# Urea-Self Powered Biosensors: A Predictive Evolutionary Model for Human Energy Harvesting

**DOI:** 10.3390/s23198180

**Published:** 2023-09-29

**Authors:** Javad Mohebbi Najm Abad, Afshin Farahbakhsh, Massoud Mir, Rasool Alizadeh, Amin Hekmatmanesh

**Affiliations:** 1 Department of Computer Engineering, Quchan Branch, Islamic Azad University, Quchan 9479176135, Iran; javad.mohebi@gmail.com; 2 Department of Chemical Engineering, Quchan Branch, Islamic Azad University, Quchan 9479176135, Iran; afshin.farahbakhsh@gmail.com; 3 Department of Mechanical Engineering, Quchan University of Technology, Quchan 9477177870, Iran; massoudmir@qiet.ac.ir; 4 Department of Mechanical Engineering, Quchan Branch, Islamic Azad University, Quchan 9479176135, Iran; rasoolalizadeh86@gmail.com; 5 Laboratory of Intelligent Machines, LUT University, 53850 Lappeenranta, Finland

**Keywords:** self-powered biosensor, urease enzyme, energy harvesting, evolutionary strategy

## Abstract

The objective of this study is to create a reliable predictive model for the electrochemical performance of self-powered biosensors that rely on urea-based biological energy sources. Specifically, this model focuses on the development of a human energy harvesting model based on the utilization of urea found in sweat, which will enable the development of self-powered biosensors. In the process, the potential of urea hydrolysis in the presence of a urease enzyme is employed as a bioreaction for self-powered biosensors. The enzymatic reaction yields a positive potential difference that can be harnessed to power biofuel cells (BFCs) and act as an energy source for biosensors. This process provides the energy required for self-powered biosensors as biofuel cells (BFCs). To this end, initially, the platinum electrodes are modified by multi-walled carbon nanotubes to increase their conductivity. After stabilizing the urease enzyme on the surface of the platinum electrode, the amount of electrical current produced in the process is measured. The optimal design of the experiments is performed based on the Taguchi method to investigate the effect of urea concentration, buffer concentration, and pH on the generated electrical current. A general equation is employed as a prediction model and its coefficients calculated using an evolutionary strategy. Also, the evaluation of effective parameters is performed based on error rates. The obtained results show that the established model predicts the electrical current in terms of urea concentration, buffer concentration, and pH with high accuracy.

## 1. Introduction

The harvesting of biological energy sources will play a significant role in the production of emission-free electricity. At present, the quantities of biological energies harvested are relatively small and can only be utilized for powering certain applications, such as biosensors and environmental, medical, and diagnostic sensor advancements [1]. Sweat is one of the biological resources that has potential for specific and critical applications. This bodily fluid, which is secreted as a result of metabolism or physical exertion, contains valuable compounds that could be harnessed as a raw material for generating electricity [2].

Urea, a waste compound that is typically secreted on the skin, contains a concentration of approximately 22.2 mM in sweat—about 3.6 times higher than the amount in blood serum [3]. This makes it a promising candidate for establishing an enzyme source of energy based on urea. By using bio-energies instead of conventional energies, a fundamental change in biosensor manufacturing technology can be achieved, as electrochemical biosensors require an electrical current to perform the identification process and create the initial potential difference necessary to initiate the process [1]. The emerging self-powered biosensors are based on biofuel cells (BFCs) and generate electrical energy using enzymatic reactions within the biosensor’s body [4]. This technology has the potential to revolutionize the field of electrochemical sensors, as it eliminates the need for external electrical sources, thus making biosensors more environmentally friendly and biocompatible. Urease is one of the hydrolase enzymes that catalyzes the hydrolysis of urea (the main substrate) and causes the release of ammonia and carbon dioxide. The positive charges from the released ammonia absorb free electrons and act as an anode to generate the electrical current and potential difference. This potential difference provides the energy required for the operation of electrochemical biosensors during the detection process [5].

In electrochemical processes, the conductivity of electrodes plays a critical role in enhancing the speed of electron transfer in biosensors. To improve electrode conductivity, high conductivity compounds can be used, and the surface of electrodes can be modified with conductive materials. Multi-walled carbon nanotubes are among the most common materials used to modify electrode surfaces due to their ability to create a large transfer surface with minimal obstruction, allowing for increased electrode conductivity [6].

To facilitate the transfer of electrons between the analyte and receptor on the working electrode and generate an electrical current in electrochemical biosensors, a potential difference is required. In conventional biosensors, this potential difference is supplied by an external electrical source or a chemical process [2]. However, the latest generation of biosensors—known as self-powered biosensors—incorporates a source of electrical energy within the structure of the biosensor itself. These biosensors are designed based on biochemical processes that are fully compatible with the biological environment of living organisms.

In addition to modifying electrode surfaces, the selection of suitable compounds is also crucial in improving the performance and efficiency of biosensors. Many researchers have investigated new compounds to improve enzyme stabilization and contact with the electrode. For example, Aušra Valiūnienė et al., demonstrated that the use of Prussian blue to bind and stabilize enzymes on working electrodes significantly increased the efficiency of the electron transfer generated by the enzyme reaction to the electrode surface [7].

Nanoparticles and nanocomposites have emerged as promising materials for enhancing the conductivity of electrodes in electrochemical biosensors [8,9]. Senel et al., demonstrated the significant impact of gold microneedles on the conductivity of the electrode in urea biosensors by incorporating gold nanoparticles in the form of ink on the electrode surface [10]. The use of gold nanoparticles resulted in the increased efficiency and sensitivity of the working electrode, owing to their high conductivity.

Likewise, researchers have explored the unique properties of graphene oxide to improve the structure of urea biosensors, as demonstrated by Razumiene et al. [11] and Priyannth et al. [12]. Silver nanoparticles and ZnO have also garnered significant attention for their potential to enhance conductivity [13,14,15,16]. In addition, NiO and polystyrene–sulphonate nanocomposites have also been investigated by researchers [17,18]. Recent studies have examined the use of zeolites and macroporous polypyrrole to modify and enhance the performance of urea biosensors [19,20].

This study focuses on the use of urea as a source energy for producing electricity. The modified electrode with multi-walled carbon nanotubes catalyzes an enzymatic hydrolysis process that breaks down urea into positive and negative ions, generating a potential difference that drives an electrical current. To enhance the reaction rate and improve the efficiency and reproducibility of the reaction, the urease enzyme is employed as a biological catalyst [6]. The platinum electrode is coated with multi-walled carbon nanotubes to increase its conductivity. The enzymatic reaction under investigation is the hydrolysis of urea by the enzyme urease, a substrate present in sweat [1,12]. To determine the factors and parameters that affect the enzymatic reaction of urea hydrolysis, a machine learning approach is employed. This approach enables the evaluation of a wide range of parameters and facilitates the prediction of comprehensive formulae that can improve the performance of the biosensor [4].

## 2. Self-Powered BFC-Based Biosensors

**Figure 1:**
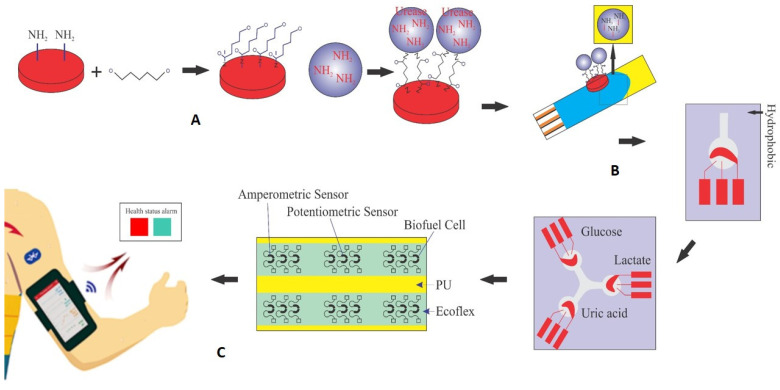
Schematic of the production process and performance of self-powered urea biosensors. (**A**) Stabilization of urease enzyme on pyrrole surface using glutaraldehyde intermediate. (**B**) The surface containing the enzyme is placed on the surface of the working electrode; the detection kit (biosensor) consists of three separate electrodes (working, counter, reference). (**C**) After fixing the components to the working electrode for each kit, they are all placed on a flexible surface such as polyurethane (PU) for the simultaneous detection and measurement of certain compounds in sweat such as uric acid (urea), glucose, or lactate.

In an electrochemical biosensor, the key components are the analyte, the receptor, and the converter. In this study, the biosensor converter utilizes electrical energy generated from the enzymatic process of urea hydrolysis. As depicted in Figure 1, the immobilization of the urease enzyme was carried out using pyrrole and polyurethane (PU) as a conductive polymer substrate, and the fabrication of the urea biosensor is shown schematically during the three stages of the manufacturing process in Figure 1A. First, a glucoaldehyde mediator is placed on the surface of the polymer as a mediator and stabilizer. The intermediate substance is connected to the substrate on one side and to the enzyme as a marker of the desired analyte on the other side, and with the formation of this bond, the enzyme is stabilized on the surface of the polymer. As seen from Figure 1B, the surface containing the enzyme is placed on the surface of the working electrode. The detection kit (biosensor) consists of three separate electrodes (working, counter, reference) and an electrical current is generated by applying a potential difference. The amount of analyte in the sample is determined by the difference between the applied and measured current. Figure 1C shows that after fixing the components on the working electrode for each kit, they are all placed on a flexible surface such as polyurethane (PU). This collection of components can be used for the simultaneous detection and measurement of some important and vital compounds in sweat such as uric acid, urea, glucose, and lactate, similar to three biosensors connected to one axis. The array can even be placed on flexible, wearable surfaces and used as sweat-based biosensors on clothing or wristbands and armbands (as shown in Figure 1).

After the stabilization of the compounds on the working electrode and complete drying at a temperature of about 25 °C, the working electrode acts as an anode and can serve as an electron transporter, generating a potential difference. Flexible and wearable kits can be achieved using electrical circuit printing technology, utilizing conductive inks that contain nanoparticles like multi-walled carbon nanotubes and silver [6,13].

## 3. Testing Method and Current Measurement

In this study, experimental data were obtained using platinum working electrodes, counter electrodes, and a silver reference electrode. The platinum electrodes have a purity of 99.95% and were prepared by a local company with qualified standards. The platinum working electrodes were polished with alumina powder (0.03 to 0.5 μg) to remove impurities and deposits on their surfaces. To improve the material stability of the electrodes, both chemical and electrochemical methods were employed to wash the electrode surfaces [9,14]. Chemical washing was performed with acetone and double-distilled water in several steps, while electrochemical washing was carried out with 1 M sulfuric acid at a voltage of −0.21 to +1.19 V and a scan speed of 50 mV/s for 10 to 15 min to ensure the cleanliness of the electrode surface.

In the first stage of preparing the working electrode, it was necessary to stabilize the pyrrole conductive polymer and the urease enzyme on the surface of the electrode. A solution containing 22 mL of 0.1 M potassium chloride, 22 mL of 0.1 M sodium chloride, and 6 mL of 0.4 M pyrrole monomer was prepared, and 0.0025 U/mL of urease enzyme was added. The solution was then stirred for 5 min using a magnetic stirrer [10]. The cleaned working electrode was immersed in the solution, and the compounds were stabilized on the electrode surface by applying a voltage in the range of −0.7 V to +1.2 V and a scan speed of 50 mV/s for 10 to 15 min and 30 cycles in the vicinity of the reference Ag/AgCl electrode using a potentiostat device. The electrode was dried at room temperature for 10 min.

During the stabilization process, 0.616 g multi-walled carbon nanotubes (diameter: 100 nm, powder, Merck, KGaA, Darmstadt, Germany) were added to the solution prior to the addition of the urease enzyme. The prepared solution was then sonicated at 25 °C for 1 h, resulting in the deposition of multi-walled carbon nanotubes on the electrode surface and the improved conductivity of the working electrode [6,13].

**Figure 2:**
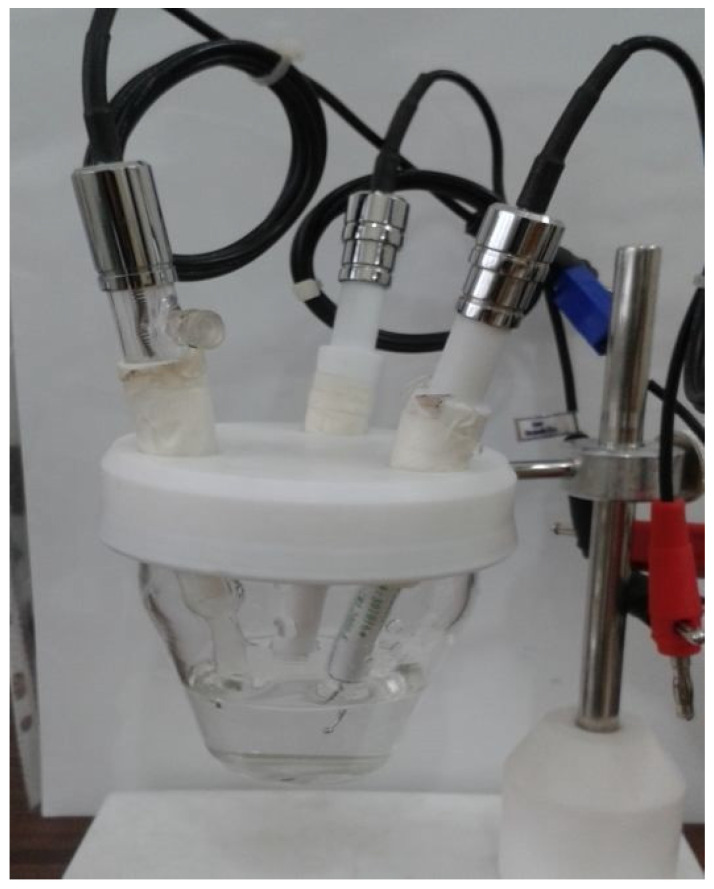
Set of electrodes connected to a potentiostat (model 7050, AMEL Company, Milano, Italy) device used to measure the amount of electrical current generated by the urea enzyme.

The working electrode, modified with multi-walled carbon nanotubes, acts as an electron producer during the electrochemical process. The Ag/AgCl reference electrode maintains a constant potential difference, while the counter electrode, made of pure platinum, acts as an electron acceptor and transmitter (see Figure 2).

Experimental data were collected to measure the generated electrical current under various conditions of urea concentration, potassium phosphate buffer concentration, and electrolyte pH. The Taguchi method was utilized to design the experiments. The concentrations of urea were 0.5, 1, and 1.5 mM, while the concentrations of potassium phosphate buffer were 0.05, 0.1, and 0.25 mM. The pH of the electrolyte was also varied at 5.5, 6, and 6.5. All solutions were employed as analytes for measuring the generated electrical current. The potentiostat device (model 7050, AMEL Company, Milano, Italy) and the Juniorassist v3 design program were used to obtain the electrical current values, which are presented in Table 1.

The Taguchi method is a commonly used experimental design technique. Instead of conducting all possible tests, a limited number of tests are performed using special orthogonal arrays to investigate the effects of variables, and the optimal conditions are predicted based on the results obtained. Taguchi’s method reduces the number of tests required by selecting orthogonal arrays from the total number of factorial experiments based on specific criteria. Although Taguchi does not guarantee that the optimal solution exists in the selected experiments, the method can determine the desired optimal conditions using calculations related to the arrays. The accuracy of the results is verified by repeating the tests under optimal conditions [21].

The platinum working electrode, on which the urease enzyme is stabilized, is immersed in various urea solutions (Table 1). Urea hydrolysis (Equations (1) and (2)) [1] occurs in the presence of the urease enzyme, resulting in the production of ammonium ions, bicarbonate ions, and hydroxides.

(1) $${NH}_{2}{CONH}_{2}+3H_{2}O\overset{urease}{\overbrace{\to }}2{NH}_{4}^{+}+HCO_{3}^{-}+{OH}^{-}$$

(2) $${NH}_{4}^{+}\overset{e}{\overbrace{\to }}{NH}_{3}+H^{+}$$

The presence of the urease enzyme on the platinum working electrode causes the hydrolysis reaction of urea (Equations (1) and (2)) [1], resulting in the production of ammonium ions, bicarbonate ions, and hydroxides. The generated ammonium ions receive electrons and are converted to ammonia, a positively charged ion in solution, thereby reducing the negative charge in the solution and creating a positive potential difference. This property serves as an energy source and current generator in electrochemical biosensors that utilize oxidizing and electron-producing enzymes, such as glucose biosensors that operate based on glucose oxidation in the presence of the glucose oxidase enzyme. The reduction peak produced by placing a working electrode in sweat activates the detection of other available compounds, such as glucose, completing the power supply cycle in self-powered BFC-based biosensors [4,21,22].

## 4. Prediction of Electrical Current Based on Taguchi Method

This paper utilizes the Taguchi method to minimize the number of experiments required and to reduce the need for further repetitions. Using this method, only 9 out of the 27 required tests are conducted, while the results for the remaining 18 experiments can be predicted. The predicted results for these experiments are in agreement with the values listed in Table 2.

Based on the findings from Table 1 and Table 2 in this study, we employed a machine learning technique to extrapolate the results to a wider range and to better represent natural samples, such as urea concentrations found in sweat. Furthermore, we derived a functional equation that correlates the variables of buffer concentration (Cb), pH, and the concentration of urea.

## 5. Presented Equation Using Evolutionary Strategy Algorithm

The article employs the Covariance Matrix Adaptation Evolution Strategy (CMA-ES) algorithm, a popular optimization method, to calculate the coefficients of the given equation as follows:

(3) $$EF=a_{0}+a_{1}{pH}^{a_{2}}+a_{3}{Cb}^{a_{4}}+a_{5}Ca$$

where EF, pH, $C_{b}$, and $C_{a}$ are the electrical current, pH buffer, urea concentration, and buffer concentration, respectively. Also, $a_{i}$(*i* = 0, …, 5) are the coefficients of the equation, which are calculated using the CMA-ES algorithm. The algorithm searches the range of [−100, 100] to identify the optimal coefficients.

The evolutionary strategy generates a set of probable solutions using a particular algorithm, evaluates each solution, and creates the next generation of solutions based on the evaluation results. The expectation is to improve the results with each successive generation until a satisfactory solution is found.

The evolutionary strategy employs a probabilistic distribution, such as the normal distribution with a mean of m and a constant step size of σ, to generate solutions. The value of m is initialized at the center of the problem path space and is subsequently determined based on the best solutions from the previous generations. The value of σ is adjusted according to the problem’s exploration needs, with an increase in σ leading to more exploration and a reduction in σ indicating a higher level of confidence in the optimal solution’s proximity. At the beginning of the algorithm, the maximum value of σ is used, and it decreases as the number of iterations increases. It is worth noting that the algorithm narrows down the search space as it approaches the optimal solution, allowing for a more detailed examination of a smaller space [23].

The CMA-ES algorithm utilizes a normal distribution to generate a fresh population, where the estimation of a covariance matrix and a mean vector is based on the current population. Through appropriate rules, the covariance matrix is continually updated to effectively guide the evolution of population creation.

The CMA-ES algorithm employs two fundamental principles to adapt the parameters of the search distribution. The first is the maximum-likelihood principle, which enhances the likelihood of candidate solutions and search procedures succeeding. To this end, CMA-ES implements an iterative principal component analysis of successful search procedures that preserves all principal axes. In this algorithm, the covariance matrix is leveraged to increase the likelihood of successful solutions instead of successful search steps.

The second principle involves evolution paths that are recorded based on the average distribution strategy during different stages. These paths provide valuable information, such as the correlation between successive steps. If the distributions develop in the same direction in successive steps, the evolutionary paths become longer. These paths are used in two ways: adapting the covariance matrix instead of successive steps, which increases the likelihood of more variance moving towards the desired solution, and in step size control direction. The purpose of step size control is to generate balanced movements based on successive distributions. Proper step control prevents premature convergence while also enabling a faster and more comprehensive exploration of the search space [24].

To utilize the CMA-ES algorithm, certain parameters require initialization. These include the mean (m) and step size (σ) values, as well as the number of population members (λ) and parents (μ) to generate each generation. Additionally, initial values for the covariance matrix (*C*), accumulation for covariance matrix (*p*_c_), and step size (*p*_σ_) must be specified. These values are typically set to:

$$C=I,\;p_{c}=0\;and\;p_{\sigma }=0,$$

where I is the identity matrix. Also, a number of other parameters are used in order to perform updates in the algorithm:

$$C_{c}\approx \frac{4}{n},\;C_{\sigma }\approx \frac{4}{n},\;C_{1}\approx \frac{2}{n^{2}},\;C_{\mu }\approx \frac{\mu_{w}}{n^{2}},\;C_{1}+C_{\mu }\leq 1,\;d_{\sigma }\;\approx 1+\sqrt{\frac{\mu_{w}}{n}},\;w_{i=1,\ldots ,\lambda },$$

such that $\mu_{w}=\frac{1}{\sum_{i=1}^{\mu }{w_{i}}^{2}}\approx 0.3\lambda$ and n is the problem dimension.

*C_c_* and *C_σ_* denote the learning rates for the cumulation of rank-one updates of the covariance matrix and step size control, respectively. Similarly, *C*_1_ and *C_μ_* represent the learning rates for rank-one and rank-μ updates, respectively.

The CMA-ES algorithm adjusts the search space in each generation by evaluating the results of the previous generation. To achieve this, it modifies the parameters μ and σ, and calculates the complete covariance matrix of the parameter space. The calculations are carried out on the top-performing solutions (μ) of the current generation, typically using 25% of the best solutions. The process consists of five steps (1 to 5) that are executed until an acceptable solution is found or a predetermined number of iterations is reached [25,26].

1. In the first step, the algorithm generates diverse solutions using the following equations:
  $$x_{i}=m+\sigma y_{i}\;,\;y_{i}=N\left(0,C\right),\;i=1,\ldots ,\lambda$$

$N\left(0,C\right)$ is the multivariate normal distribution with mean 0 and covariance matrix C.

2. Then, the evaluation of the solutions is carried out using the following relationship:
  $$f\left(x_{i}\right),\;i=1,\ldots ,\lambda$$
3. The solutions are sorted according to their evaluation. In updating the mean value (m), the number μ of the best selected solutions from the current generation is used as follows:
  $$m\leftarrow m+\sigma \bar{y},\;where\;\bar{y}=\sum_{1}^{\mu }w_{i}\;y_{i:\lambda }$$
4. The step size control is updated using the following relations:

$$p_{\sigma }\leftarrow \left(1-C_{\sigma }\right)p_{\sigma }+\sqrt{C_{\sigma }(2-C_{\sigma })\mu_{w}}C^{-\frac{1}{2}}\bar{y}$$

$$\sigma \leftarrow \sigma \;\exp(\frac{C_{\sigma }}{d_{\sigma }}(\frac{\left\|p_{\sigma }\right\|}{E\left\|N\left(0,I\right)\right\|}-1))$$

where $N\left(0,I\right)$ is the multivariate normal distribution with zero mean and unity covariance matrix.

5. Then, the covariance matrix is updated employing the following relations:
  $$p_{c}\leftarrow \left(1-C_{c}\right)p_{c}+\sqrt{C_{c}(2-C_{c})\mu_{w}}\bar{y}$$
  $$C\leftarrow \left(1-C_{1}-C_{\mu }\right)C+C_{1}p_{c}{p_{c}}^{T}+C_{\mu }\sum_{1}^{\mu }w_{i}\;y_{i:\lambda }{y_{i:\lambda }}^{T}$$

The result of using the CMA-ES algorithm to find the coefficients of Equation (3) is the following answer vector:

$$\vec{a}=<2.43222\;,\;0.30658\;,\;0.866196\;,-3.3403528\;,\;0.0580531,-0.044922>$$

## 6. Results and Discussion

**Figure 3:**
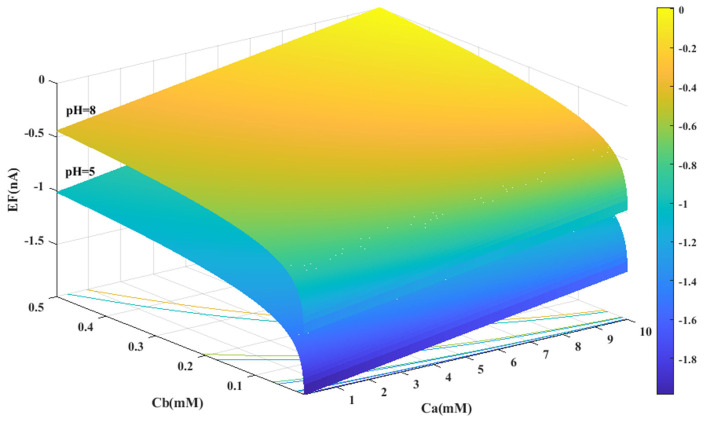
Predicted value of generated electrical current in urea biosensor based on the proposed model in terms of $C_{a}$ and $C_{b}$ for two different pH levels.

**Figure 4:**
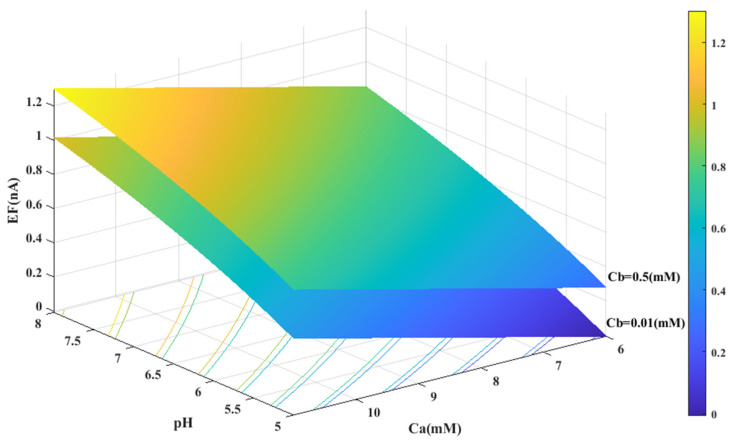
Predicted value of generated electrical current in urea biosensor based on the proposed model in terms of $C_{a}$ and pH for two different values of $C_{b}$.

**Figure 5:**
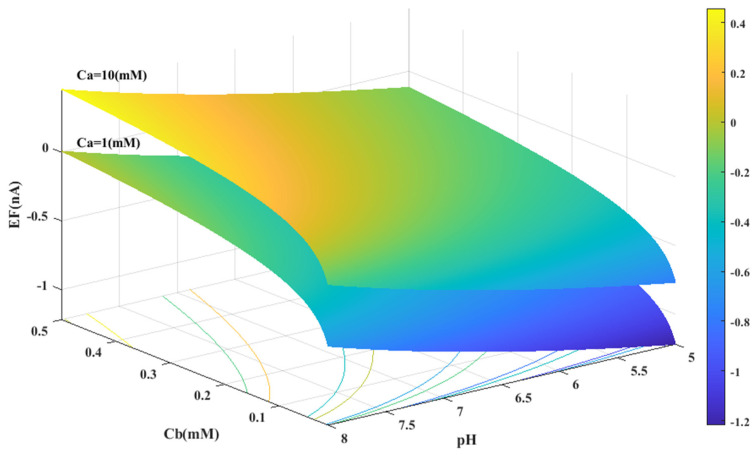
Predicted value of generated electrical current in urea biosensor based on the proposed model in terms of $C_{b}$ and pH for two different values of $C_{a}$.

Based on the experimental data and a generalized equation derived from artificial intelligence, the impact of three distinct parameters, namely, the pH of the analyte solution, the concentration of urea (C_a_ (mM)), and the concentration of phosphate buffer (C_b_ (mM)), on the magnitude of the electrical current (EF (µA)) generated during the urea hydrolysis reaction is examined and assessed. Figure 3, Figure 4 and Figure 5 illustrate the results of this investigation within a reasonable and realistic range of pH values and concentrations.

Figure 3 displays the concentration of urea and buffer on the horizontal axes, and the range of generated current on the vertical axis. According to Figure 3, the amount of electrical current in alkaline conditions (pH = 8) is higher than in acidic conditions (pH = 5). It can be concluded that the alkaline environment can provide more efficiency for the performance of urea biosensors and urea hydrolysis will be more complete in these conditions.

In all cases, the parameter intervals are selected to reflect the actual system conditions. Two pH values, one in the acidic range (pH = 5) and the other in the alkaline range (pH = 8), were examined to investigate the effect of pH on the reaction. The urea concentration range was set between 0 and 10 mM, and the buffer concentration range was set between 0 and 0.5 mM. Figure 4 and Figure 1C were also created with a fixed vertical axis (produced electrical current) and the pH-Ca and pH-Cb horizontal axes, respectively, with intermediate levels of Ca and Cb.

**Figure 6:**
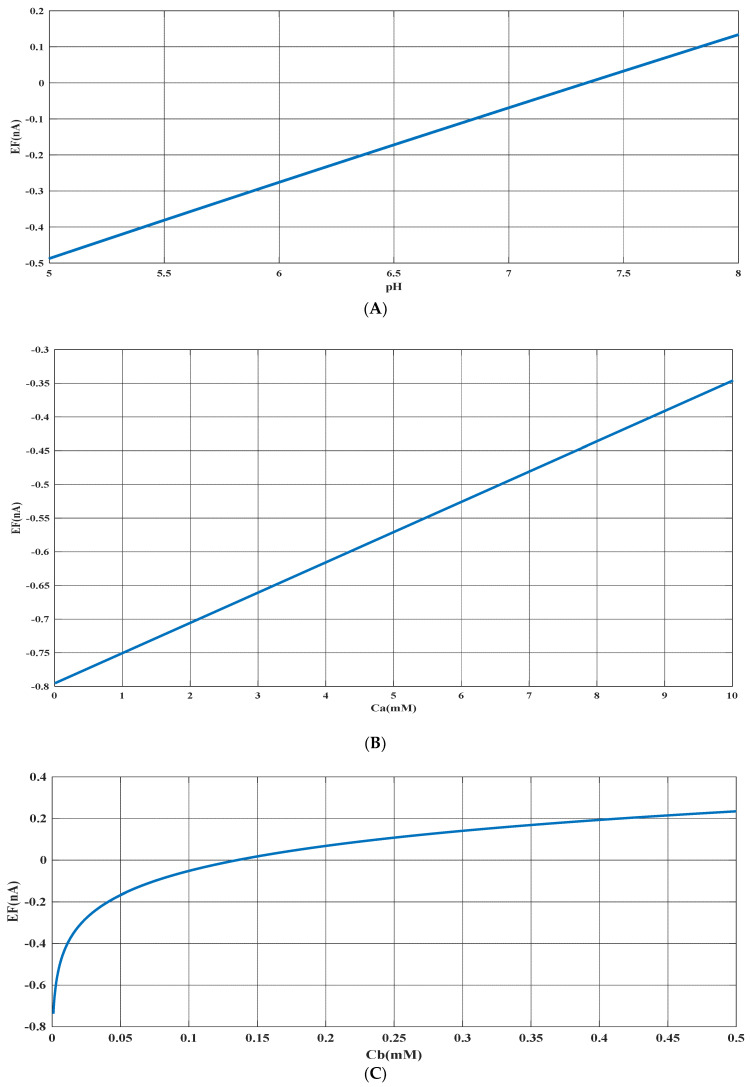
(**A**) Predicted value of generated electrical current in urea biosensor based on the proposed model in terms of pH for constant values of $C_{a}=10\;milimolar$ and $C_{b}=0.5\;mM$. (**B**) Predicted value of generated electrical current in urea biosensor based on the proposed model in terms of $C_{a}$ for constant values of $C_{b}=0.5\;mM$ and pH=8. (**C**) Predicted value of generated electrical current in urea biosensor based on the proposed model in terms of $C_{b}$ for constant values of $C_{a}=10\;mM$ and pH=8.

The results indicate that the urea hydrolysis reaction is more efficient in an alkaline environment, as demonstrated by the production of more electrical current at pH = 8 compared to pH = 5 and the increase in the amount of produced current with an increase in pH (as depicted in Figure 3 and Figure 6A). This suggests that the urease enzyme is capable of catalyzing more urea molecules in an alkaline environment and generating an electrical current. Consequently, achieving higher efficiency necessitates an alkaline environment in the analyte solution.

Based on Figure 3, Figure 4, Figure 5 and Figure 6B,C, there appears to be a notable correlation between Ca and Cb. Increasing the buffer concentration has a positive effect on the reaction, as evidenced by the steep slope in the generated current as the buffer concentration increases up to approximately 0.5 mM, indicating optimal reaction conditions. However, after reaching this point, the effect of buffer concentration on the reaction rate decreases and remains constant, indicating a uniform trend. This may suggest that the increased buffer concentration creates a more favorable environment for optimal enzyme activity, increasing the likelihood of urea binding to the enzyme’s active site and accelerating the generation of electrical current at the start of the reaction.

Moreover, it can be inferred from the diagram that increasing the buffer concentration up to 0.5 mM accelerates the performance of the urease enzyme in the reaction and the production of an electrical current.

Through a detailed analysis of Figure 4 and Figure 6B, it is evident that an increase in the concentration of urea in the analyte solution leads to a proportional increase in the generated electrical current. This trend is observed across both pH values and is maintained at an optimal buffer concentration of 0.5 mM with a linear slope. The observation that urea concentration has a uniform impact on the generated electrical current, irrespective of the pH and buffer concentration, highlights the importance of maintaining stability in pH and buffer concentration during the enzymatic reaction. Therefore, to achieve optimal reaction conditions and obtain accurate measurement results, it is essential to maintain a stable, alkaline pH and an optimal buffer concentration of 0.5 mM in the analyte or enzyme-activating solution.

Based on an artificial intelligence analysis of various factors, it is evident that an increase in urea concentration can enhance the production of electrical current, provided that the environmental conditions of the reaction remain stable. In particular, pH and buffer concentration have a significant impact on the process, and this research indicates that elevating these variables can lead to an increase in electrical current. However, it is important to maintain the buffer concentration at the optimal value of 0.5 mM to achieve optimal results.

It is worth noting that these factors are meticulously monitored and controlled during the reaction to prevent any adverse effects. Furthermore, the stability of these conditions in body sweat makes it an ideal substrate for the production of urea-based bio-electricity, which can generate high-efficiency electrical current for various biological applications, including self-powered biosensors.

## 7. Conclusions

The results of this study demonstrate that utilizing the urea hydrolysis reaction in the presence of a urease biocatalyst can produce satisfactory levels of electrical current. This approach has two significant applications: constructing a urea biosensor and developing biological electricity. In a urea biosensor, changes in electrical current can be used to detect and quantify urea in various environments. Additionally, the enzymatic reaction of urea and the resulting electrical current can be used as a power source for self-powered biosensors. The obtained electrical current value of 22.2 mM is greater than the minimum required value of 0.5 mM needed to power a biosensor. Furthermore, the study demonstrates that under optimal Cb, Ca, and pH parameters, the maximum predicted electrical current generated by artificial intelligence is 0.215 µA. This level of electrical current is sufficient to activate and operate BFCs/self-powered biosensors.

## Figures and Tables

**Table 1:** Generated electrical current values from urea-based energy for different conditions of urea concentration, buffer, and pH according to Taguchi test design.

Test Number	C_a_ (Urea Concentration (mM))	C_b_ (Buffer Concentration (mM))	pH (Buffer)	EF (Electrical Current (nA))
1	0.5	0.25	5.5	0.67
2	1	0.1	5.5	0.87
3	1.5	0.05	5.5	0.9
4	1	0.25	6	0.775
5	1.5	0.1	6	0.775
6	0.5	0.05	6	1.05
7	1.5	0.25	6.5	0.85
8	0.5	0.1	6.5	0.82
9	1	0.05	6.5	1.175

**Table 2:** Predicted values of electrical current based on Taguchi method.

Test Number	C_a_ (Urea Concentration (mM))	C_b_ (Buffer Concentration (mM))	pH (Buffer)	EF (Electrical Current (nA))
10	0.5	0.05	5.5	1.17
11	1	0.05	5.5	1.07
12	0.5	0.1	5.5	0.93
13	1.5	0.1	5.5	0.73
14	1	0.25	5.5	0.93
15	1.5	0.25	5.5	0.8
16	0.5	0.25	6	0.86
17	1.5	0.25	6	0.67
18	0.5	0.1	6	0.78
19	1	0.1	6	0.71
20	1	0.05	6	0.9
21	1.5	0.05	6	0.77
22	0.5	0.25	6.5	1
23	1	0.25	6.5	0.91
24	1	0.1	6.5	0.82
25	1.5	0.1	6.5	0.71
26	0.5	0.05	6.5	1.14
27	1.5	0.05	6.5	0.89

## Data Availability

Not applicable.

## References

[1] Lv J., Yin L., Chen X., Jeerapan I., Silva C.A., Li Y., Le M., Lin Z., Wang L., Trifonov A. (2021). Wearable Biosupercapacitor: Harvesting and Storing Energy from Sweat. Adv. Funct. Mater..

[2] Raza T., Qu L., Khokhar W.A., Andrews B., Ali A., Tian M. (2021). Progress of Wearable and Flexible Electrochemical Biosensors with the Aid of Conductive Nanomaterials. Front. Bioeng. Biotechnol..

[3] Keller R.W., Bailey J.L., Wang Y., Klein J.D., Sands J.M. (2016). Urea transporters and sweat response to uremia. Physiol. Rep..

[4] Zhou M., Wang J. (2012). Biofuel Cells for Self-Powered Electrochemical Biosensing and Logic Biosensing: A Review. Electroanalysis.

[5] Zeng X., Peng R., Fan Z., Lin Y. (2021). Self-powered and wearable biosensors for healthcare. Mater. Today Energy.

[6] Lotfi L., Farahbakhsh A., Aghili S. (2018). Modification of Glucose Oxidase biofuel cell by multi-walled carbon nanotubes. Nanophotonics Australasia 2017.

[7] Valiūnienė A., Kavaliauskaitė G., Virbickas P., Ramanavičius A. (2021). Prussian blue based impedimetric urea biosensor. J. Electroanal. Chem..

[8] Jeerapan I., Sonsa-ard T., Nacapricha D. (2020). Applying Nanomaterials to Modern Biomedical Electrochemical Detection of Metabolites, Electrolytes, and Pathogens. Chemosensors.

[9] Bahrololoomi A., Bilan H.K., Podlaha E.J. (2022). Electrodeposited Ni-Fe onto Glassy Carbon for the Detection of Methylene Blue. J. Electrochem. Soc..

[10] Senel M., Dervisevic M., Voelcker N.H. (2019). Gold microneedles fabricated by casting of gold ink used for urea sensing. Mater. Lett..

[11] Razumiene J., Gureviciene V., Sakinyte I., Rimsevicius L., Laurinavicius V. (2020). The Synergy of Thermally Reduced Graphene Oxide in Amperometric Urea Biosensor: Application for Medical Technologies. Sensors.

[12] Baabu P.R.S., Gumpu M.B., Nesakumar N., Rayappan J.B.B., Kulandaisamy A.J. (2020). Electroactive Manganese Oxide–Reduced Graphene Oxide Interfaced Electrochemical Detection of Urea. Water Air Soil Pollut..

[13] Singh A.K., Singh M., Verma N. (2019). Electrochemical preparation of Fe_3_O_4_/MWCNT polyaniline nanocomposite film for development of urea biosensor and its application in milk sample. J. Food Meas. Charact..

[14] Liu J., Moakhar R.S., Perumal A.S., Roman H.N., Mahshid S., Wachsmann-Hogiu S. (2020). An AgNP-deposited commercial electrochemistry test strip as a platform for urea detection. Sci. Rep..

[15] Eghbali M., Farahbakhsh A., Rohani A., Pour A.N. (2015). Urea biosensor based on immobilization of urease on ZnO nanoparticles. Orient. J. Chem..

[16] Zhou F., Jing W., Xu Y., Chen Z., Jiang Z., Wei Z. (2019). Performance enhancement of ZnO nanorod-based enzymatic glucose sensor via reduced graphene oxide deposition and UV irradiation. Sens. Actuators B Chem..

[17] Tyagi M., Tomar M., Gupta V. (2019). Enhanced electron transfer properties of NiO thin film for the efficient detection of urea. Mater. Sci. Eng. B.

[18] Uzunçar S., Meng L., Turner A.P., Mak W.C. (2021). Processable and nanofibrous polyaniline:polystyrene-sulphonate (nano-PANI:PSS) for the fabrication of catalyst-free ammonium sensors and enzyme-coupled urea biosensors. Biosens. Bioelectron..

[19] Soldatkina O.V., Kucherenko I.S., Pyeshkova V.M., Dudchenko O.Y., Kurç B.A., Dzyadevych S.V. (2019). Development of electrochemical biosensors with various types of zeolites. Appl. Nanosci..

[20] Hosseinian M., Najafpour G., Rahimpour A. (2019). Amperometric urea biosensor based on immobilized urease on polypyrrole andmacroporous polypyrrole modified Pt electrode. Turk. J. Chem..

[21] Ramezani Azghandi O., Farahbakhsh A. (2015). The obtain optimum production conditions for Glucose Oxidase biosensor using software Qualtek-4. Int. J. Nano Dimens. (IJND).

[22] Yang W., Han W., Gao H., Zhang L., Wang S., Xing L., Zhang Y., Xue X. (2018). Self-powered implantable electronic-skin for *in situ* analysis of urea/uric-acid in body fluids and the potential applications in real-time kidney-disease diagnosis. Nanoscale.

[23] Mesgarpour M., Wongwises S., Alizadeh R., Mohammadiun H., Mohammadiun M. (2021). The comparative investigation of three approaches to modeling the natural convection heat transfer: A case studyon conical cavity filled with Al_2_O_3_ nanoparticles. J. Taiwan Inst. Chem. Eng..

[24] Abad J.M.N., Alizadeh R., Fattahi A., Doranehgard M.H., Alhajri E., Karimi N. (2020). Analysis of transport processes in a reacting flow of hybrid nanofluid around a bluff-body embedded in porous media using artificial neural network and particle swarm optimization. J. Mol. Liq..

[25] Mesgarpour M., Abad J.M.N., Alizadeh R., Wongwises S., Doranehgard M.H., Jowkar S., Karimi N. (2022). Predicting the effects of environmental parameters on the spatio-temporal distribution of the droplets carrying coronavirus in public transport—A machine learning approach. Chem. Eng. J..

[26] Mohammadiun M., Mohammadiun H., Alizadeh R., Mesgarpour M., Younesian A., Jowkar S. (2021). The effect of variable temperature and location on relative thermal conductivity (RTC) on the heat pipe in the presence of AL_2_O_3_ nanoparticles: Numerical and optimization approaches. J. Taiwan Inst. Chem. Eng..

